# High Rate of Inappropriate Antibiotics in Patients with Hematologic Malignancies and Pseudomonas aeruginosa Bacteremia following International Guideline Recommendations

**DOI:** 10.1128/spectrum.00674-23

**Published:** 2023-06-27

**Authors:** Mariana Chumbita, Pedro Puerta-Alcalde, Lucrecia Yáñez, Maria Angeles Cuesta, Anabelle Chinea, Ignacio Español-Morales, Pascual Fernandez-Abellán, Carlota Gudiol, Pedro González-Sierra, Rafael Rojas, José María Sánchez-Pina, Irene Sánchez Vadillo, Miguel Sánchez, Rosario Varela, Lourdes Vázquez, Manuel Guerreiro, Patricia Monzo, Carlos Lopera, Tommaso Francesco Aiello, Oliver Peyrony, Alex Soriano, Carolina Garcia-Vidal

**Affiliations:** a Hospital Clínic de Barcelona-IDIBAPS, Universitat de Barcelona, Barcelona, Spain; b Hospital Universitario Marqués de Valdecilla, Santander, Spain; c Hospital Universitario Virgen de la Victoria, Málaga, Spain; d Hospital Universitario Ramón Y Cajal, Madrid, Spain; e Hospital Clínico Universitario Virgen de la Arrixaca, Murcia, Spain; f Hospital General Universitario de Alicante, Alicante, Spain; g Hospital Universitario de Bellvitge, Institut Català d'Oncologia, IDIBELL, l'Hospitalet de Llobregat, Barcelona, Spain; h Hospital Universitario Virgen de las Nieves, Granada, Spain; i Hospital Universitario Reina Sofia, Córdoba, Spain; j Hospital Universitario 12 de Octubre, Madrid, Spain; k Hospital Universitario La Paz, Madrid, Spain; l Hospital Universitario de A Coruña, Coruña, Spain; m Complejo Asistencial Universitario de Salamanca, Salamanca, Spain; n Hospital Universitario y Politécnico La Fe, Valencia, Spain; o Centro de Investigación Biomédica en Red (CIBER) de Enfermedades Infecciosas, Barcelona, Spain; p Emergency Department, Hôpital Saint-Louis, Assistance Publique-Hôpitaux de Paris, Paris, France; University at Albany

**Keywords:** neutropenia, bacteremia, *P. aeruginosa*, mortality, empirical antibiotic treatment

## Abstract

Optimal coverage of Pseudomonas aeruginosa is challenging in febrile neutropenic patients due to a progressive increase in antibiotic resistance worldwide. We aimed to detail current rates of resistance to antibiotics recommended by international guidelines for P. aeruginosa isolated from bloodstream infections (BSI) in patients with hematologic malignancies. Secondarily, we aimed to describe how many patients received inappropriate empirical antibiotic treatment (IEAT) and its impact on mortality. We conducted a retrospective, multicenter cohort study of the last 20 BSI episodes caused by P. aeruginosa in patients with hematologic malignancies from across 14 university hospitals in Spain. Of the 280 patients with hematologic malignancies and BSI caused by P. aeruginosa, 101 (36%) had strains resistant to at least one of the β-lactam antibiotics recommended in international guidelines, namely, cefepime, piperacillin-tazobactam, and meropenem. Additionally, 21.1% and 11.4% of the strains met criteria for MDR and XDR P. aeruginosa, respectively. Even if international guidelines were followed in most cases, 47 (16.8%) patients received IEAT and 66 (23.6%) received inappropriate β-lactam empirical antibiotic treatment. Thirty-day mortality was 27.1%. In the multivariate analysis, pulmonary source (OR 2.22, 95% CI 1.14 to 4.34) and IEAT (OR 2.67, 95% CI 1.37 to 5.23) were factors independently associated with increased mortality. We concluded that P. aeruginosa-causing BSI in patients with hematologic malignancies is commonly resistant to antibiotics recommended in international guidelines, which is associated with frequent IEAT and higher mortality. New therapeutic strategies are needed.

**IMPORTANCE** Bloodstream infection (BSI) caused by P. aeruginosa is related with an elevated morbidity and mortality in neutropenic patients. For this reason, optimal antipseudomonal coverage has been the basis of all historical recommendations in the empirical treatment of febrile neutropenia. However, in recent years the emergence of multiple types of antibiotic resistances has posed a challenge in treating infections caused by this microorganism. In our study we postulated that P. aeruginosa-causing BSI in patients with hematologic malignancies is commonly resistant to antibiotics recommended in international guidelines. This observation is associated with frequent IEAT and increased mortality. Consequently, there is a need for a new therapeutic strategy.

## INTRODUCTION

Bloodstream infection (BSI) are the most frequent infectious complication in patients with hematologic malignancies, being related with high morbidity and mortality (1, 2). One of the most severe and difficult-to-treat bacteria causing BSI in this population is Pseudomonas aeruginosa. It is related to an elevated morbidity and mortality (3, 4). For this reason, optimal coverage of this pathogen has been one of the most formidable challenges in febrile neutropenic guidelines (5, 6).

Nowadays, proper empirical coverage of this pathogen has become even more problematic due to a progressive increase in multidrug-resistant (MDR) and extensively drug-resistant (XDR) P. aeruginosa isolates worldwide (7, 8). Despite this marked epidemiological change, the most up-to-date antibiotic guidelines for patients with hematologic malignancies and neutropenia continue recommending an empirical β-lactam treatment that includes cefepime, piperacillin-tazobactam, and meropenem (6, 9, 10). Our hypothesis is that most physicians still use these antibiotics as empirical treatment, given the observed high rates of inappropriate empirical antibiotic treatment (IEAT) and more elevated mortality.

We aimed to describe the current rates of resistance to β-lactam antibiotics recommended in international guidelines for P. aeruginosa isolated from BSI in patients with hematologic malignancies. Secondarily, we aimed to describe how many patients received IEAT and its impact on mortality.

## RESULTS

### Cohort and BSI characteristics.

We analyzed 280 episodes of BSI caused by P. aeruginosa in patients with hematologic malignancies. Table 1 summarizes the demographic and clinical characteristics of the cohort. The most frequent hematologic malignancies were acute leukemia (36.8%) and non-Hodgkin lymphoma (26.4%). Furthermore, 114 (40.7%) patients had received a hematopoietic stem cell transplant, mostly allogeneic (69, 60.5%). A total of 170 (60.9%) patients were neutropenic at BSI onset. Most BSI were endogenous (86, 30.7%), followed by catheter-related and pulmonary source (55, 19.6% and 48, 17.1%, respectively).

**TABLE 1:** Demographic and clinical characteristics of the cohort

Characteristic	N = 280 (%)
Demographic	
Male sex	176 (63.1)
Median (IQR) age, yrs	60 (50–68)
Hematological disease	
Acute leukemia	103 (36.8)
Non-Hodgkin lymphoma	74 (26.4)
Multiple myeloma	39 (13.9)
Myelodysplastic syndrome	20 (7.1)
Hodgkin's lymphoma	13 (4.6)
Chronic lymphocytic leukemia	11 (3.9)
Bone-marrow aplasia	4 (1.4)
Others	16 (5.7)
Hematopoietic stem cell transplant	114 (40.7)
Allogenic	69 (24.6)
Neutropenia	170 (60.9)
Source of BSI	
Endogenous/unknown	86 (30.7)
Catheter-related	55 (19.6)
Pulmonary	48 (17.1)
Skin and soft-tissue infection	23 (8.2)
Abdominal	21 (7.5)
Urinary tract	19 (6.8)
Others	15 (5.3)

### Antimicrobial resistance profile and the appropriateness of empirical antibiotics.

**FIG 1:**
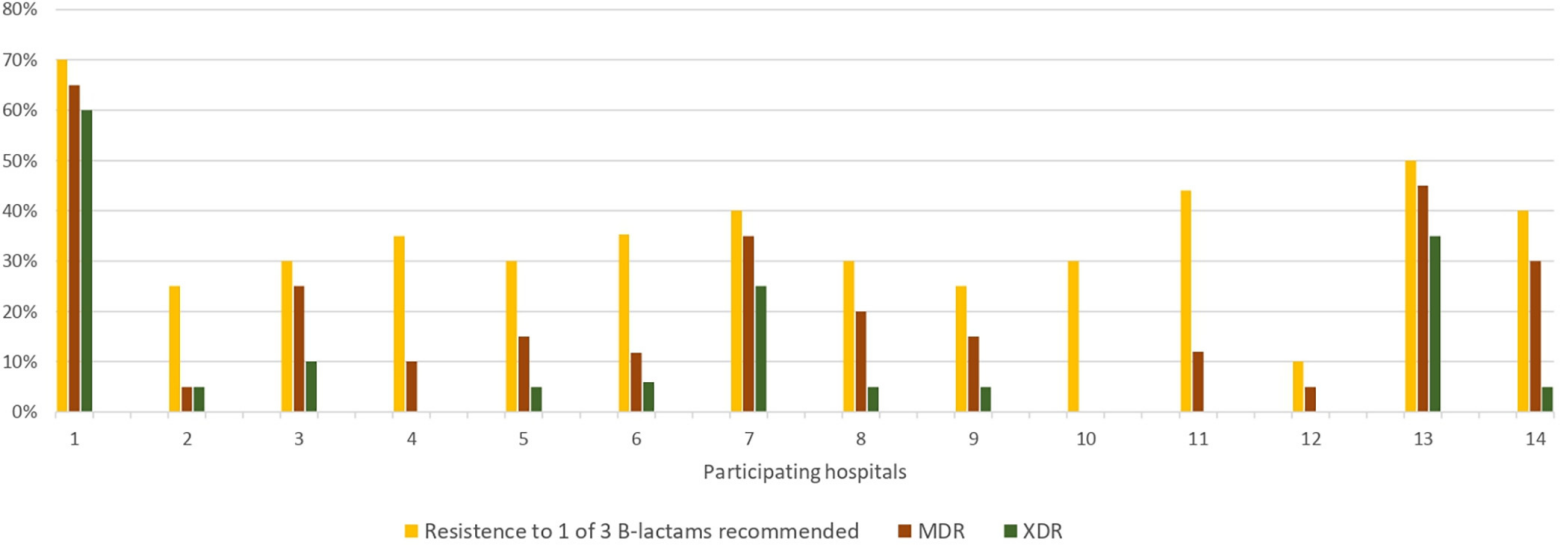
Rates of resistance to at least 1 of the β-lactam antibiotics recommended in the international guidelines, and rates of multi drug-resistant (MDR) and extensively drug-resistant (XDR) P. aeruginosa isolates, across the different hospitals.

Of all isolated P. aeruginosa, 101 (36.1%) were resistant to at least one of the β-lactam antibiotics recommended in international guidelines, namely, cefepime, piperacillin-tazobactam, or meropenem (Table 2). The highest rate of resistance was to quinolones (82, 29.3%) and the lowest to amikacin (41, 14.6%). A total of 21.1% and 11.4% of the strains met criteria for MDR and XDR P. aeruginosa, respectively. Figure 1 shows the antimicrobial resistance distribution across the different participating centers.

**TABLE 2:** Resistance profiles among bloodstream infections caused by Pseudomonas aeruginosa in hematologic patients with febrile neutropenia^*a*^

Antibiotic	N = 280 (%)
Quinolones	82 (29.3)
Piperacillin-tazobactam	61 (21.8)
Cefepime	72 (25.7)
Meropenem	70 (25)
Amikacin	41 (14.6)
MDR-P. aeruginosa	59 (21.1)
XDR-P. aeruginosa	32 (11.4)
Resistance to at least 1 of the β-lactam antibiotics recommended in the international guidelines	101 (36.1)

a EUCAST MIC breakpoints R> (mg/L): Ciprofloxacin: >0.5; Piperacillin-tazobactam: >16; Cefepime: >8; Meropenem: >8; Amikacin: >16. Abbreviations: MDR, multidrug resistant; XDR, extensively drug resistant.

The most frequently administered empirical antibiotic was meropenem (114, 40.7%), followed by piperacillin-tazobactam (103, 36.8%) and cefepime (45, 16.1%). An empirical antibiotic combination was administered in 68 (24.3%) patients, mainly with amikacin or colistin (17.1% and 5.7%, respectively).

Although international guidelines were followed in 262 (94%) of cases, 47 (16.8%) patients received IEAT, and 66 (23.6%) received inappropriate β-lactam empirical antibiotic treatment. Inappropriate β-lactam therapy was documented in 32% (36 of 114) of patients receiving meropenem, 20% (21/103) of those receiving piperacillin-tazobactam, and 16% (7 of 45) of those receiving cefepime. When the isolated P. aeruginosa was resistant to at least one of the antimicrobials recommended by the guidelines, IEAT increased significantly (43.6% versus 1.7%, *P* < 0.001). Likewise, IEAT was more common in those episodes caused by MDR-P. aeruginosa (42.4% versus 10%, *P* < 0.001) and XDR-P. aeruginosa (40.6% versus 13.7%, *P* < 0.001).

### Outcomes.

Overall, 30-day mortality was 76 (27.1%). Table 3 shows the univariate and multivariate analyses of risk factors for mortality. In the univariate analysis, mortality was higher in BSI episodes from a pulmonary source (42.8% versus 23.9%, *P* = 0.009) and episodes caused by either MDR-P. aeruginosa (42.4% versus 23.1%, *P* = 0.003) or an isolate that was resistant to at least one of the β-lactam antibiotics recommended by the guidelines (37.6% versus 21.2%, *P* = 0.003). Likewise, mortality was more elevated among those patients receiving IEAT (48.9% versus 22.7%, *P* < 0.001). Conversely, mortality was significantly lower when patients received a combination of two active antibiotics (12.8% versus 29.5%, *P* = 0.030).

**TABLE 3:** Univariate and multivariate analysis for 30-day mortality among 280 episodes of bacteriemia due to Pseudomonas aeruginosa

Risk factor	Survivors N = 204 (%)	Deaths N = 76 (%)	Univariate OR (95% CI)	*P* value	Multivariate OR (95% CI)	*P* value
Male sex	123 (69.9)	53 (30.1)	1.98 (0.85–2.63)	0.159		
Over 70 yrs	41 (65.1)	22 (34.9)	1.62 (0.88–2.95)	0.115		
Acute Leukemia	76 (73.8)	27 (26.2)	0.92 (0.53–1.60)	0.790		
Allogeneic hematopoietic stem cell transplantation	49 (71)	20 (29)	1.13 (0.61–2.06)	0.692		
Neutropenia	121 (71.2)	49 (28.8)	1.23 (0.71–2.15)	0.458		
Pulmonary source	29 (58)	21 (42)	2.30 (1.21–4.36)	0.009	2.27 (1.14–4.34)	0.019
Unknown source	66 (75.9)	21 (24.1)	0.79 (0.44–1.42)	0.448		
Combination therapy with an aminoglycoside	37 (77)	11 (22.9)	0.76 (0.36–1.58)	0.469		
Combination therapy with colistin	10 (62.5)	6 (37.5)	1.66 (0.58–4.74)	0.337		
Active combination therapy^*a*^	34 (87.2)	5 (12.8)	0.35 (0.13–0.93)	0.030	0.40 (0.148–1.11)	0.080
Quinolone resistance	55 (67.1)	27 (32.9)	1.49 (0.85–2.62)	0.161		
Piperacillin-tazobactam resistance	39 (63.9)	22 (36.1)	1.72 (0.94–3.16)	0.076		
Cefepime resistance	47 (65.3)	25 (34.7	1.63 (0–91−2.92)	0.093		
Meropenem resistance	41 (58.6)	29 (41.4)	2.45 (1.37–4.36)	0.002	1.58 (0.80–4.49)	0.179
Amikacin resistance	23 (56.1)	18 (43.9)	2.44 (1.23–4.84)	0.009	1.38(0.62–3.09)	0.424
Resistance to at least 1 of the 3 antibiotics recommended by neutropenia guidelines^*b*^	63 (62.4)	38 (37.6)	2.23 (1.30–3.83)	0.003	0.85 (0.32–2.27)	0.751
Multidrug resistant P. aeruginosa	34 (57.6)	25 (42.4)	2.46 (1.34–4.48)	0.003	1.24 (0.45–3.42)	0.665
Inappropriate empirical antibiotic therapy	24 (51.1)	22 (48.9)	3.25 (1.70–6.22)	<0.001	2.67 (1.37–5.23)	0.004

a Beta-lactam and aminoglucoside or colistin combinations are active *in vitro.*

b Piperacillin-tazobactam, meropenem, or cefepime/ceftazidime.

In the multivariate analysis, pulmonary sources of BSI (OR 2.27, 95% CI 1.14 to 4.34) and IEAT (OR 2.67, 95% CI 1.37 to 5.23) were independently associated with increased mortality. The goodness of fit of the multivariate model was assessed using the Hosmer-Lemeshow test (0.819). The discriminatory power of the score, as evaluated by the area under the receiver operating characteristic curve, was 0.692 (95% CI 0.622 to 0.762). This figure demonstrated a good ability to predict 30-day mortality.

## DISCUSSION

The current study describes the resistance to empirical antibiotic regimen in a multicenter cohort of patients with hematologic malignancies and P. aeruginosa bacteremia. The most important finding is that over a third of BSIs were caused by strains resistant to the anti-pseudomonal β-lactams recommended as empirical treatment in international guidelines. It is troubling to show that almost 20% of P. aeruginosa BSI episodes received IEAT and rose to almost 45% in those cases with resistance to at least one of the β-lactam antibiotics recommended by the guidelines and/or in MDR isolates. These results are in concordance with those previously published in similar populations in other countries (11, 12).

This observation is not surprising given the current epidemiology of multiresistance in P. aeruginosa strains worldwide. In our cohort, over 20% and 10% of the isolates met criteria for MDR and XDR, respectively. This, however, varied widely among centers. Previous studies conducted in immunocompromised hosts (4, 13–16) also reported rates of MDR isolates between 23% and 33%. Similar data have also been published in nonneutropenic patients from a large multicenter cohort from Spain; 26.2% of isolates were classified as MDR and 17.3% as XDR (17). More importantly, however, resistance to each of the different beta-lactam antibiotics recommended by international guidelines reached more than 20% (9, 10).

This information is extremely important, since IEAT has been clearly related to increased mortality in these patients (18–20). In line with previous evidence, IEAT was an independent risk factor for a more elevated mortality rate in the current cohort. The likelihood of death in those patients who received incorrect empirical antibiotics doubled.

Considering the high rates of IEAT and related increased mortality, new strategies are needed to improve patients’ prognosis. Different studies have attempted to describe risk factors for MDR-P. aeruginosa BSI to reduce the probability of IEAT (11, 14, 21). However, mostly described risk factors are general and unspecific, and result in low applicability to real-life scenarios. In the coming years, though, via analysis of multiple variables in real-time, big data, and artificial intelligence algorithms may prove useful in predicting the specific risk of an MDR isolate (22, 23). These algorithms could guide the employment of new rapid diagnostic tests for multidrug resistance. The role that surveillance cultures can play in identifying patients at high risk of multidrug resistance remains to be investigated.

Another potential strategy to improve the prognosis of these patients may be antibiotic combination therapies. We have previously described that amikacin combination was an independent protective factor in patients with neutropenia, BSI, and septic shock (24). However, in this paper and other similar publications, combination therapy was only beneficial when both amikacin and the β-lactam were active (13, 19, 25). Conversely, when only amikacin was active, mortality was exceedingly high. These results suggest that the potential benefit of amikacin does not widen the antimicrobial spectrum; instead, it confers a synergistic effect alongside the β-lactam.

Given all of the aforementioned information, it is paramount to ensure the appropriateness of the initial β-lactam. In recent years, new anti-pseudomonal β-lactams have become available, namely, ceftolozane-tazobactam, ceftazidime-avibactam, and cefiderocol. Even in the current scenario of high MDR-P. aeruginosa prevalence, these antibiotics remain active against more than 90% of the isolated Pseudomonas (17). Additionally, a few studies have already shown that these new antibiotics could be even more effective in patients with hematologic malignancies (26–29). In a study conducted at the MD Anderson Cancer Center, empirical treatment with ceftolozane-tazobactam was associated with better clinical outcomes than standard of care (cefepime, meropenem, or piperacillin-tazobactam) in patients with hematologic malignancies and febrile neutropenia (26). However, in this same study, only 6 of the 100 enrolled patients had a microbiologically documented infection. In another study, Bergas et al. (28) retrospectively compared ceftolozane-tazobactam against other antibiotics in patients with hematologic malignancies and P. aeruginosa BSI, in which most episodes (91%) were due to MDR isolates. In this study, treatment with ceftolozane-tazobactam was significantly associated with both a lower need for mechanical ventilation and reduced mortality. This drug has also shown good efficacy in real-life patients with severe ESBL-producing Enterobacterales infections (30).

Considering these results, ceftolozane-tazobactam should be considered an initial empirical antibiotic therapy in patients with hematologic malignancies in those centers with a current resistance rate of more than 10% against one of the three classical antipseudomonal antibiotics in P. aeruginosa infections, as well as with a low incidence of carbapenemase-producing Enterobacterales infections. In hospitals with a high incidence of carbapenemase-producing Enterobacterales infections in patients with hematologic malignancies, ceftazidima/avibactam appears to be a suitable option. Early antibiotic deescalation is possible and safe after clinicians rule out P. aeruginosa BSI within the first 24 to 48 h (31, 32).

The strength of this study includes a description of a current, prospective, and multicenter cohort from different regions of Spain and a large number of patients. However, there are some limitations that must be acknowledged. We performed a noninterventional study. Consequently, decisions regarding empirical antibiotics varied across the centers and respective local epidemiology is probably determined by the endemicity of some MDR pseudomonal strains. Also, the attending physician decided the management of intermediate antibiotic breakpoints. In addition, we included all university hospitals. It is likely that the IEAT rates are higher in centers which are not university hospitals. It is mandatory to be familiar with the ecology of each region and create personalized treatment protocols that respond to patients' needs.

In conclusion, we found that over a third of P. aeruginosa strains-causing BSI in patients with hematologic malignancies are resistant to the antibiotics recommended in international guidelines. In this scenario, IEAT was frequent and independently associated with increased mortality. Novel strategies to address this situation are needed.

## MATERIALS AND METHODS

### Setting, study population, and design.

This is a retrospective study of a multicenter, observational, and prospective cohort, which was conducted across 14 tertiary hospitals in Spain. In October 2019, we collected the last 20 episodes of BSI caused by P. aeruginosa in adult (>18 years) patients with hematologic malignancies from each center. We recorded the following data: age and sex, baseline disease, source of BSI, antimicrobial susceptibility profile, empirical antibiotic treatments, and 30-day mortality.

This study was approved by the Ethics Committee Board of Hospital Clinic (HCB/2021/0157).

### Definitions.

Patients with febrile neutropenia were defined as those who had a single temperature measurement of >38.3°C or that of >38.0°C sustained over a 1-h period, and an absolute neutrophil count of ≤500 cells/mm^3^ (5). The source of bacteremia was defined according to standard Centers for Disease Control (CDC) criteria (33).

Empirical antibiotic therapy was defined as that administered at BSI onset. Combination therapy was defined as administering more than one antibiotic empirically. IEAT was reported when the empirical antibiotic therapy did not include at least one *in vitro* active antibiotic. Additionally, P. aeruginosa was classified as MDR or XDR per prior consolidated definitions (34). Mortality was defined as death by any cause within the first 30 days of BSI onset.

### Microbiological methods.

Blood samples were processed using the Bactec 9240 system or Bactec FX system (Becton, Dickinson Microbiology Systems), with a 5-day incubation period. Isolates were identified by standard techniques. Antimicrobial susceptibility testing was performed using either a microdilution system (Microscan WalkAway Dade Behring, West Sacramento, CA or Phoenix system, Becton, Dickinson, Franklin Lakes, NJ) or the Etest (AB Biodisk, Solna, Sweden/bioMérieux, Mercy l’Etoile, France).

To define susceptibility or resistance to these antimicrobial agents, EUCAST version 9.0 of the EUCAST clinical breakpoint tables was used (35).

### Statistical analysis.

Categorical variables were described by counts and percentages, whereas continuous variables were expressed as means and standard deviations (SD) or medians and interquartile ranges (IQRs). The Chi-squared Pearson test and Student’s t-test were used to compare categorical and continuous variables, respectively. A multivariate regression model (step-forward procedure) was used to identify independent risk factors for mortality. The multivariate analysis included all statistically significant variables in the univariate analysis. The goodness of fit of the multivariate model was assessed by the Hosmer-Lemeshow test and the area under the receiver operating characteristic curve. The threshold for statistical significance was defined as a two-tailed *P* < 0.05. All analyses were performed using SPSS software (version 25.0; SPSS, Inc., Chicago, IL).
